# The effect of incentives on intertemporal choice: Choice, confidence, and eye movements

**DOI:** 10.3389/fpsyg.2022.989511

**Published:** 2022-11-03

**Authors:** Xing-Lan Yang, Si-Tan Chen, Hong-Zhi Liu

**Affiliations:** ^1^Department of Social Psychology, Zhou Enlai School of Government, Nankai University, Tianjin, China; ^2^Laboratory of Behavioral Economics and Policy Simulation, Nankai University, Tianjin, China

**Keywords:** incentive, intertemporal choice, decision confidence, eye-tracking, loss

## Abstract

Despite various studies examining intertemporal choice with hypothetical rewards due to problematic real reward delivery, there remains no substantial evidence on the effect of the incentives on the decision confidence and cognitive process in intertemporal choice and no comprehensive exploration on the loss domain. Hence, this study conducts an eye-tracking experiment to examine the effect of incentive approach and measure participants' decision confidence using a between-subject design in both gain and loss domains. Results replicated previous findings which show incentives do not affect intertemporal choice in the gain domain. In contrast, in the loss domain, participants in the incentivized group were more likely to choose the larger-later options than those in the non-incentivized group. Furthermore, the decision confidence and the mean fixation duration differed between the incentivized and non-incentivized groups in both gain and loss domains. These findings allow for a better understanding of the effect of incentives on intertemporal choice and provide valuable information for the design of incentives in future intertemporal experiments.

## 1. Introduction

The concept of intertemporal choice, which involves balancing rewards and costs that arise at various times, is ubiquitous and widespread (Loewenstein and Prelec, 1992; Frederick et al., 2002). Examples of intertemporal choice include buying (e.g., investing in a new laptop now or hold off till the price has considerably dropped for a few months), health (e.g., overeat now for temporary satisfaction or stick to a healthy diet for a good body), and saving (e.g., spend the salary immediately or save for retirement) habits and behaviors (Fisher, 2021). The most popular approach to investigating intertemporal decisions has been eliciting choices between smaller-sooner (SS) and larger-later (LL) monetary amount (e.g., Kirby, 1997; Weber et al., 2007; Hardisty and Weber, 2009; Scholten and Read, 2010; Cubitt et al., 2017; Calluso et al., 2019). Intertemporal choice can be characterized by the classical economic theory of exponential discounting (Samuelson, 1937) and the hyperbolic discounting model (Ainslie, 1975; Frederick et al., 2002) where an individual's patience is described by a single hyperbolic discount rate (*k*-value) which links to intertemporal decisions. Compared to a higher *k*-value, which suggests steeper delay discounting and more impatient choices, a lower *k*-value shows slower discounting of future outcomes and is thus more likely to make patient decisions.

Although incentives are generally considered to be essential in economic experiments, substantial research examined intertemporal choice with hypothetical rewards (e.g., Estle et al., 2007; Rao and Li, 2011; Read et al., 2013; Dai and Busemeyer, 2014; Liu et al., 2021a). This may be due to the cost of using real rewards being prohibitive and the temporal delays for giving monetary rewards can be lengthy, making real reward delivery problematic (Johnson and Bickel, 2002; Bickel et al., 2009). Several studies have examined the difference between hypothetical and real rewards in intertemporal choice and showed that incentives have no significant effect on participants' temporal discounting (Johnson and Bickel, 2002; Madden et al., 2004). For instance, using the within-subject design, no systematic difference was found in observed discounting rates between hypothetical and real rewards (Johnson and Bickel, 2002; Madden et al., 2003). This was replicated in a between-subject design experiment (Madden et al., 2004) where subsequent research also found that the incentives have no significant effect on steady-state intertemporal outcomes (Lagorio and Madden, 2005). The evidence seemed to excuse the researchers who did not include incentives in their intertemporal experiments because of the cost—where the sentence, “we used hypothetical rewards because previous studies have shown that there was no difference between real and hypothetical intertemporal outcomes” was usually present.

However, several aspects of the effect of the incentive approach on intertemporal choice remain unclear. First, previous research has focused on the difference between hypothetical and real intertemporal rewards, but did not examine loss outcomes. Intertemporal choice has been shown to be asymmetric between gain and loss frames (Thaler, 1981) and the subjective valuation of a delayed loss has been reduced less sharply than the subjective assessment of a delayed gain (Frederick et al., 2002; Jiang and Liu, 2021). Time preferences for negative experiences were also independent of time preferences for rewards (Harris, 2012). It is suspected that there might be asymmetric effect of incentives on intertemporal choice in gain and loss domains. Therefore, further examining the effect of incentives on intertemporal losses is necessary.

Second, the effect of incentives on decision confidence remains unexplored. A participant's confidence in the qualities of a judgment is referred to as “decision confidence” (Peterson and Pitz, 1988) which is an aspect of metacognition (Fleming et al., 2012). This confidence can be measured using retrospective judgments where participants self-report the uncertainty in their decisions, in the form of subjective accuracy, for various decision tasks (Fleming and Lau, 2014). Compared with the dichotomous output of choice, the quantitative estimation of confidence tends to be more sensitive to incentives (Sandberg et al., 2010; Wierzchon et al., 2012). For instance, Lak et al. (2014) showed that orbitofrontal cortex inactivation decreases decision confidence without affecting decision accuracy. Thus, the incentives may not affect intertemporal choice but may influence decision confidence.

Third, the effect of incentives on the information process underlying intertemporal choice also lacks substantial evidence. Recently, the focus of decision-making research has shifted from being purely behaviorist on choice outcomes to process-tracing approaches on decision processes (Glaholt and Reingold, 2011). Among these process-tracing methodologies, eye-tracking technique is effective in recording the cognitive processes involved in making decisions (Ashby et al., 2016). Researchers have explored the cognitive process underlying intertemporal choice using this technique (Franco-Watkins et al., 2016; Reeck et al., 2017; Amasino et al., 2019; Marini et al., 2020; Fisher, 2021; Liu et al., 2021a). However, some of these studies did not use incentives (e.g., Franco-Watkins et al., 2016; Liu et al., 2021a). Thus, examining the effect of incentives on the cognitive process during intertemporal choice seems necessary.

Given the abovementioned questions, the present study further examined the effect of incentives on intertemporal choice. We examined the impact of the real and hypothetical intertemporal outcomes using a between-subject design in both gain and loss domains, along with measuring participants' confidence in their decisions. Using eye-tracking technology, the effect of incentives on eye-tracking measures reflecting the cognitive process during intertemporal choice was examined.

This study is exploratory in nature and has no strong hypotheses on the effect of the incentive approach on intertemporal choice. We computed the following behavioral indicators in the experiment considering the following: (1) Proportion of choosing LL options as a larger proportion of choosing LL options indicates lower temporal discounting and a higher level of patience; (2) Response time given its capacity to be utilized to differentiate between intuitive and deliberate decisions (Rubinstein, 2007). Processes that are intuitive can be carried out quicker than those that are deliberate (Krajbich et al., 2015); and (3) Decision confidence where after each intertemporal choice, participants were prompted to express how confident they were in their choice.

The following eye-tracking measures were also computed: Total dwell time (TDT), Mean fixation duration (MFD), Outcome-gaze-proportion (OGP), and the search measure index (SMI). With the first measure, the total dwell time of all the regions of interest (ROIs) is a crucial measure in the field of eye movement research (Stewart et al., 2015). Total dwell time is correlated to fixation count which is the number of fixations in a trial. The higher the value of TDT, the more time the participants spent looking at a region of interest, and the more attention was paid to this region. Research on eye movements during reading, for instance, indicates that TDT rises as text complexity does (Rayner et al., 2006). For the second measure, the average amount of time spent on a single fixation during a decision, or mean fixation duration, can indicate the intensity of cognitive effort (Velichkovsky, 1999; Horstmann et al., 2009; Amblee et al., 2017). MFD is a measure of the amount of time that participants spend thinking about the information they are fixating on, and the difficulty of information extraction or the level of interest in the visual stimuli may be indicated by a longer mean fixation duration (Wang et al., 2014). As a result, a longer mean fixation length indicates higher cognitive effort level. For OGP, its value measures the percentage of time spent to focus on an intertemporal option's outcome attribute and reflects the decision weight on the outcome attribute (Franco-Watkins et al., 2016; Amasino et al., 2019; Zhou et al., 2021). Last, the SMI value is an index that measures how much of a search is alternative-wise or attribute-wise. It depends on the difference between the observed alternative-wise and attribute-wise saccades (Böckenholt and Hynan, 1994). This index is frequently used in research on eye movements in decision-making to assess the general search direction of information acquisition (Su et al., 2013; Liu et al., 2021b; Zhou et al., 2022).

## 2. Methods

### 2.1. Participants

G*Power software (version 3.1.9.2) (Faul et al., 2007) calculated that a sample size of 128 participants would provide 80% power to detect a medium effect (Cohen's *d* = 0.5) using the *t*-test (two-tailed). A total of 150 college students (53% female, *M*_*age*_ = 20.9 ± 2.4) were recruited from a university's human subjects pool as participants. The participants were randomly assigned to either the incentivized (*N* = 75) or the non-incentivized group (*N* = 75). For their involvement, each participant got 25 RMB (roughly 3.8 USD), and those in the incentivized group received or paid two additional delayed or immediately amount determined by their performance in the experiment. All of the participants gave their prior written informed consent and had normal or corrected-to-normal eyesight. The study was approved by the institutional review board of the university.

### 2.2. Apparatus

A 17-inch LCD panel with a display resolution of 1,024 × 768 pixels and refresh rate of 60 Hz was used to display the stimuli. During the trial, participants reacted by pressing the keyboard. The participant's eyes were 60 cm away from the screen, subtending a visual angle of 35° horizontally and 28° vertically at this distance. EyeLink 1000 plus (SR Research Ltd., Ontario, Canada) eye trackers with 1,000 Hz sample rates were used to monitor the participants' eye movements. A chin rest was also employed to minimize head movements. Since both eyes are fixed on the same area, only one eye needed to be recorded. The software Experiment Builder (version 2.1.512) was used to gather experimental data.

### 2.3. Stimuli

Four delay values (now, 5, 10, and 20 days) and four outcome values (1, 5, 10, and 20 RMB) were combined to generate 36 pairs of intertemporal options with gains (see Table S1 in Supplementary materials: https://osf.io/prcbm/). Each pair of options contained one of the SS and LL options and no option was dominated by the other. A total of 36 pairs of options with losses were similarly generated. That is, four delay values (now, 5, 10, and 20 days) and four outcome values (–1, –5, –10, and –20 RMB) were combined to generate 36 pairs of intertemporal options with losses. Therefore, each participant completed 144 trials. The accurate fixation of values was ensured by the (horizontal/vertical) center-to-center spacing being larger than 5° between any two values, making it difficult for the peripheral recognition of a nearby value (Rayner, 1998, 2009).

### 2.4. Task

Participants were randomly assigned to either the incentivized or the non-incentivized condition. In each condition, participants completed two tasks: the gain and the loss task each consisting of two blocks with each block involving the same 36 pairs of intertemporal options. Therefore, participants completed 144 trials. In both tasks, the participants were asked to choose their preferred options between a pair of options (i.e., SS and LL options) and had unlimited time to decide. In the first condition, the participants were informed that one of their trials in each task would be randomly chosen at the end of the experiment and treated as a real choice. In the second condition, participants were not instructed with the information about incentivization.

The order of the tasks was also counterbalanced. Half the participants completed the gain task first, while the other half completed the loss task. The location of attribute values (i.e., delay or outcome) was balanced. In each task, the placement of attributes was counterbalanced across the two blocks. Half the participants saw the outcome as the top number in the first block, and the other half saw the delay as the top number in the first block. The location of SS and LL options were also counterbalanced. In half of the trials, the SS options were shown on the left and in the other half of the trials, the LL options were shown of the left. The options were presented in randomized order for each participant in each block.

### 2.5. Procedure

The participants were told about the experiment and were given a brief description of the equipment after providing their consent. They then went through a conventional 5-point calibration and validation procedure before recording began. The highest validation error in the visual angle was at 0.5 degrees. After the initial calibration, the participants experienced four practice trials to become accustomed to the manner in which each task was presented.

**Figure 1:**
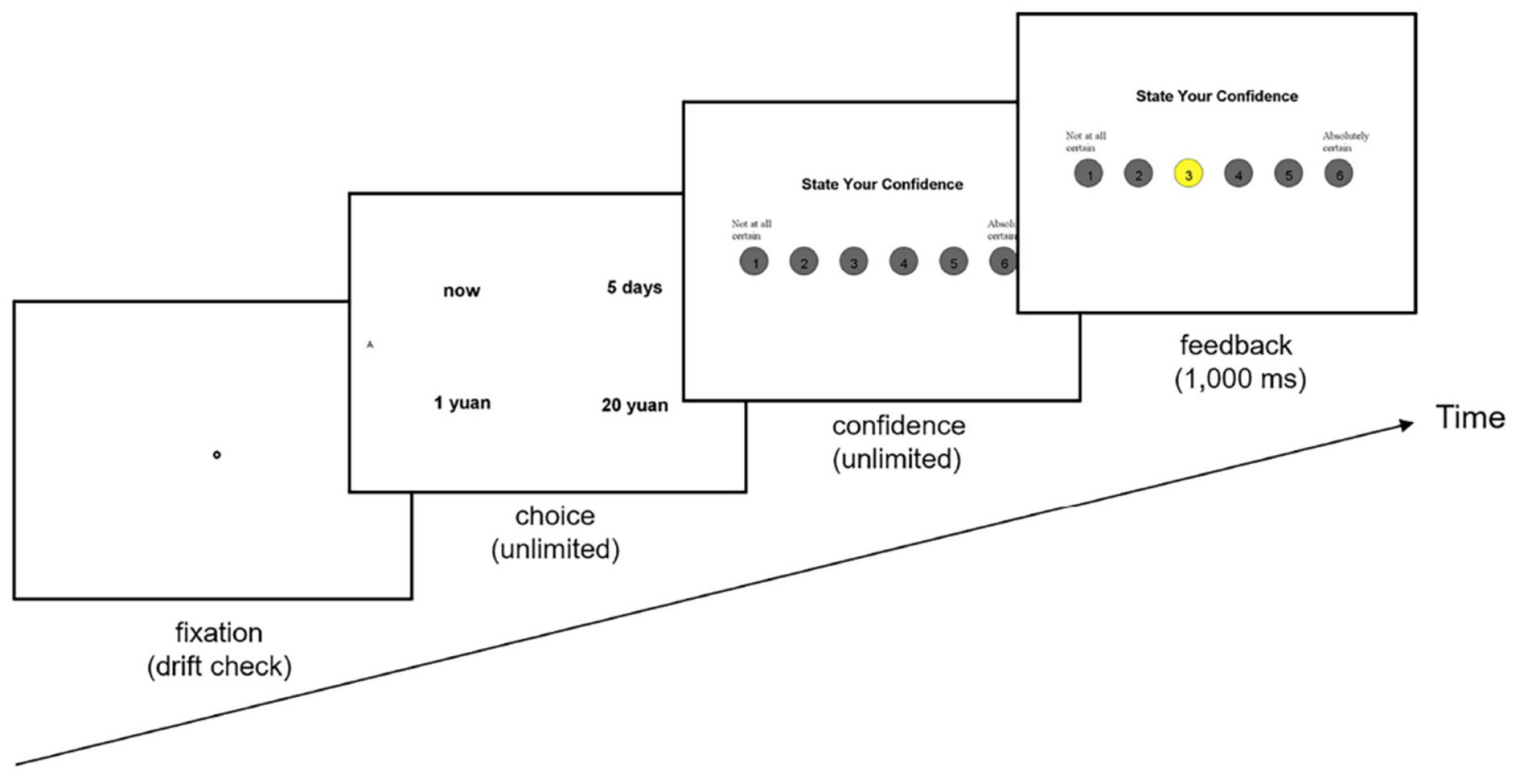
Trial procedure and timing in the experiment.

A fixation disc was displayed in the middle of the screen at the start of each trial, which also acted as an eye tracker drift check. The participants pressed the spacebar to view the alternatives after registering a fixation on the disc. Pressing “F” to select the choice on the left or “J” to select the option on the right gave participants an unlimited amount of time to make their decision. Then, using a scale of 1 (representing “Not at all certain”) to 6 (representing “Absolutely certain”), the participants stated how confident they were in their decision. A feedback screen was displayed for 1,000 ms after the participants reported their levels of confidence. The trial procedure and timing is shown in Figure 1.

### 2.6. Data analysis

**Preprocessing of eye-tracking data**. A software called EyeLink Data Viewer (version 4.2.1) was used to evaluate the choice page's eye movement data (SR Research, Ontario, Canada). Around each piece of information (i.e., the outcomes and delays), four non-overlapping, similarly sized rectangular ROIs (15.8° × 11.2° viewing angle) were established. Fixations were defined as intervals of relatively fixed gaze occurring in between saccades and those which lasted less than 50 ms were disregarded from the analyzes.

**Eye-tracking measures**. The following previously mentioned eye-tracking measures were computed.

(1) The *total dwell time* (TDT) was defined as the total amount of fixation durations during the course of a trial. The TDT was log-transformed in the data analysis.

(2) The *mean fixation duration* (MFD) was calculated by dividing the TDT by the number of fixations. The values of MFD are sensitive to cognitive effort (Zhou et al., 2022) or the complexity level of information processing (Velichkovsky, 1999; Velichkovsky et al., 2002).

(3) The *outcome-gaze-proportion* (OGP) provides an index of the amount of time spent focusing on an intertemporal option's outcome feature (Franco-Watkins et al., 2016; Amasino et al., 2019; Zhou et al., 2021) and was calculated using the following:


(1) $$\begin{array}{l} OGP=\frac{Gaze\;Duration\;to\;Outcome\;Attributes}{Gaze\;Duration\;to\;All\;Attributes} \end{array}$$

(4) The *search measure index* (SMI) is an index that measures the degree to which the search direction is alternative-wise or attribute-wise (Böckenholt and Hynan, 1994) and was calculated using the following:


(2) $$\begin{array}{l} SMI=\frac{\sqrt{N}\left[\left(\frac{AD}{N}\right)\left(r_{a}-r_{d}\right)-\left(D-A\right)\right]}{\sqrt{A^{2}\left(D-1\right)+D^{2}\left(A-1\right)}} \end{array}$$

where *r*_*a*_ and *r*_*d*_ are the number of alternative-wise transitions and attribute-wise transitions, respectively, and *N* is the number of total transitions. Here, *A* and *D* stand for the number of choices and the number of attributes, respectively (i.e., *A* = 2, *D* = 2). SMI's negative value implies a search that is primarily attribute-wise, while its positive value indicates one that is primarily alternative-wise (Pachur et al., 2013).

**Statistical analyzes**. Bayesian statistical techniques as well as traditional Null Hypothesis Significance Testing (ANOVA, *t*-test) were employed to evaluate the data. We concentrated on Bayes Factors (*BF*s) which quantified the probability of observed data given a specific hypothesis. The default 0.707-width Cauchy prior developed by the *jamovi* (version 2.2) software (Şahin and Aybek, 2019) was applied in all analyzes. We used the terminologies “weak” (for *BF* values between 1 and 3), “moderate” (for *BF* values between 3 and 10), and “strong”(for *BF* values between 10 and 30) as proposed by Wagenmakers et al. (2018) and van Doorn et al. (2021).

## 3. Results

Overall, 8 out of the 21,600 trials were discarded from analyzes due to eye-tracking failures. The dependent variables were averaged in gain and loss tasks for each participant. No other data (e.g., the outliers) were excluded from the analyzes herein.

### 3.1. Behavioral results

#### 3.1.1. Choice

**Figure 2:**
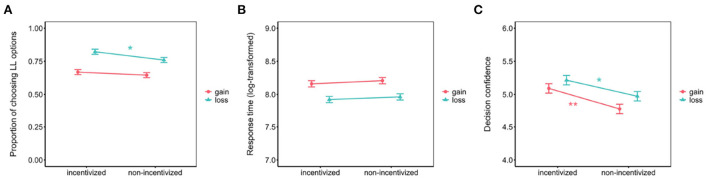
Behavioral results of **(A)** proportion of choosing LL options, **(B)** response time, and **(C)** decision confidence in the present study. Error bars represent 95% CI. ^*^p < 0.05, ^**^p < 0.01.

We calculated the proportion of choosing LL options in gain and loss tasks for each participant. A 2 (incentivize: incentivized, non-incentivized) × 2 (task: gain, loss) ANOVA was conducted with the proportion of choosing LL options as dependent variable. Results revealed a significant effect of task [*F*_(1, 148)_ = 72.30, *p* < 0.001, $\eta_{p}^{2}$ = 0.33], indicating that the proportion of choosing LL options in the gain task (*M* = 65.7%, 95% CI = [62.9%, 68.5%]) was significantly lower than that in the loss task (*M* = 79.1%, 95% CI = [76.6%, 81.7%]). The main effect of incentivize was not significant, *F*_(1, 148)_ = 3.77, *p* = 0.054, $\eta_{p}^{2}$ = 0.03 along with the interaction of incentive and task [*F*_(1, 148)_ = 1.47, *p* = 0.227, $\eta_{p}^{2}$ = 0.01]. Planned *t*-tests also showed no significant difference between incentivized (*M* = 66.9%, 95% CI = [63.0%, 70.9%]) and non-incentivized conditions (*M* = 64.5%, 95% CI = [60.6%, 68.5%]) in gain trials [*t*_(148)_ = 0.85, *p* = 0.398, Cohen's *d* = 0.14, *BF*_(01)_ = 4.09], indicating moderate evidence for the null hypothesis. However, the proportion of choosing LL options was higher in the incentivized condition (*M* = 82.3%, 95% CI = [78.6%, 85.9%]) than that in the non-incentivized condition (*M* = 76.0%, 95% CI = [72.4%, 79.7%]) in loss trials [*t*_(148)_ = 2.37, *p* = 0.019], Cohen's *d* = 0.39, *BF*_(10)_ = 2.25, which indicate weak evidence for the alternative hypothesis as shown in Figure 2A.

#### 3.1.2. Response time

Response times (the total time spent by a participant before to making an intertemporal decision, after which it was log-transformed) were examined with a 2 (incentivize) × 2 (task) ANOVA. The results revealed a significant effect of task [*F*_(1, 148)_ = 71.15, *p* < 0.001, $\eta_{p}^{2}$ = 0.33], indicating that the response times in the gain task (*M* = 8.18, 95% CI = [8.13, 8.24]) were significantly longer than those in the loss task (*M* = 7.94, 95% CI = [7.87, 8.02]). The main effect of incentivize was not significant [*F*_(1, 148)_ = 0.52, *p* = 0.471, $\eta_{p}^{2}$ = 0.004] and the interaction of incentive and task was not significant [*F*_(1, 148)_ = 0.02, *p* = 0.896, $\eta_{p}^{2}$ = 0.00]. Planned *t*-tests also showed no significant difference between incentivized and non-incentivized conditions in gain trials [*t*_(148)_ = 0.89, *p* = 0.373, Cohen's *d*, *BF*_(01)_ = 3.94] or loss trials [*t*_(148)_ = 0.51, *p* = 0.610, Cohen's *d* = 0.08, *BF*_(01)_ = 5.05]. Figure 2B illustrates these findings.

#### 3.1.3. Confidence

Decision confidence were also examined with a 2 (incentivize) × 2 (task) ANOVA. The results revealed a significant effect of task [*F*_(1, 148)_ = 13.83, *p* < 0.001, $\eta_{p}^{2}$ = 0.09], indicating that the confidence in the gain task (*M* = 4.94, 95% CI = [4.84, 5.03]) was significantly lower than that in the loss task (*M* = 5.09, 95% CI = [4.99, 5.20]). The main effect of incentive was also significant [*F*_(1, 148)_ = 9.25, *p* = 0.003, $\eta_{p}^{2}$ = 0.06]. However, the interaction of incentive and task was not significant [*F*_(1, 148)_ = 0.64, *p* = 0.427, $\eta_{p}^{2}$ = 0.004]. Planned *t*-tests showed significant difference between incentivized (*M* = 5.09, 95% CI = [4.96, 5.22]) and non-incentivized (*M* = 4.78, 95% CI = [4.65, 4.91]) conditions in gain trials [*t*_(148)_ = 3.32, *p* = 0.001, Cohen's *d* = 0.54, *BF*_(10)_ = 24.68] (strong evidence). They also showed significant difference between incentivized (*M* = 5.22, 95% CI = [5.07, 5.37]) and non-incentivized (*M* = 4.97, 95% CI = [4.82, 5.12]) conditions in loss trials [*t*_(148)_ = 2.27, *p* = 0.025, Cohen's *d* = 0.37, *BF*_(10)_ = 1.83], indicating weak evidence as exhibited in Figure 2C.

### 3.2. Eye-tracking measures

#### 3.2.1. TDT

**Figure 3:**
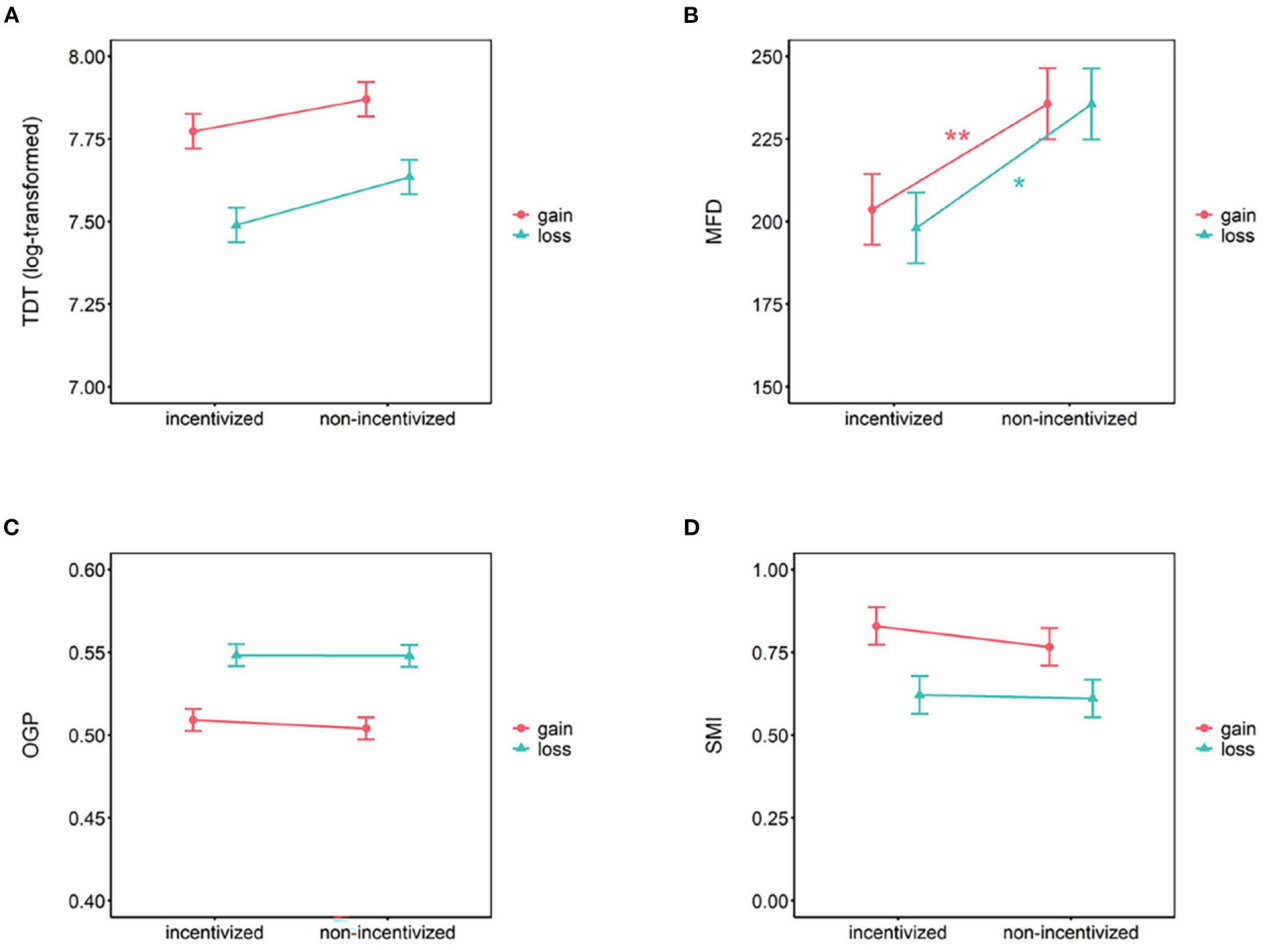
Eye-tracking measures results of **(A)** TDT, **(B)** MFD, **(C)** OGP, and **(D)** SMI in the present study. Error bars represent 95% CI. ^*^p < 0.05, ^**^p < 0.01.

A 2 (incentivize) × 2 (task) ANOVA was conducted with TDT as dependent variable. The results revealed a significant effect of task [*F*_(1, 148)_ = 64.24, *p* < 0.001, $\eta_{p}^{2}$ = 0.30], indicating that the TDT in the gain task (*M* = 7.82, 95% CI = [7.76, 7.88]) was significantly greater than that in the loss task (*M* = 7.56, 95% CI = [7.48, 7.65]). The main effect of incentivize was not significant [*F*_(1, 148)_ = 3.32, *p* = 0.070, $\eta_{p}^{2}$ = 0.02] and the interaction of incentive and task was not significant as well, *F*_(1, 148)_ = 0.54, *p* = 0.463, $\eta_{p}^{2}$ = 0.004. Planned *t*-tests also showed no significant difference between incentivized and non-incentivized conditions in gain trials [*t*_(148)_ = 1.58, *p* = 0.116, Cohen's *d* = 0.26, *BF*_(01)_ = 1.81] or loss trials [*t*_(148)_ = 1.71, *p* = 0.089, Cohen's *d* = 0.28, *BF*_(01)_ = 1.49]. Figure 3A illustrates these findings.

#### 3.2.2. MFD

A 2 (incentivize) × × 2 (task) ANOVA was conducted with MFD as dependent variable. The main effect of task was not significant [*F*_(1, 148)_ = 0.55, *p* = 0.461, $\eta_{p}^{2}$ = 0.004], along with the interaction of incentive and task [*F*_(1, 148)_ = 0.51, *p* = 0.476, $\eta_{p}^{2}$ = 0.003]. The main effect of incentivize was significant [*F*_(1, 148)_ = 5.63, *p* = 0.019, $\eta_{p}^{2}$ = 0.04]. Planned *t*-tests showed significant difference between incentivized (*M* = 204 ms, 95% CI = [186, 221]) and non-incentivized (*M* = 236 ms, 95% CI = [218, 253]) conditions in gain trials [*t*_(148)_ = 2.60, *p* = 0.010, Cohen's *d* = 0.42, *BF*_(10)_ = 3.72]. They also showed significant difference between incentivized (*M* = 198 ms, 95% CI = [173, 223]) and non-incentivized (*M* = 236 ms, 95% CI = [211, 260]) conditions in loss trials [*t*_(148)_ = 2.14, *p* = 0.034, Cohen's *d* = 0.35, *BF*_(10)_ = 1.42]. Figure 3B illustrates these findings.

#### 3.2.3. OGP

A 2 (incentivize) × 2 (task) ANOVA was conducted with OGP as dependent variable. The results revealed a significant effect of task [*F*_(1, 148)_ = 43.73, *p* < 0.001, $\eta_{p}^{2}$ = 0.23], indicating that the OGP in the gain task (*M* = 0.507, 95% CI = [0.501, 0.512]) was significantly lower than that in the loss task (*M* = 0.548, 95% CI = [0.536, 0.560]). The main effect of incentive was not significant [*F*_(1, 148)_ = 0.17, *p* = 0.684, $\eta_{p}^{2}$ = 0.001] and the interaction of incentive and task was not significant as well [*F*_(1, 148)_ = 0.10, *p* = 0.757, $\eta_{p}^{2}$ = 0.001]. Planned *t*-tests showed no significant difference between incentivized and non-incentivized conditions in gain trials [*t*_(148)_ = 0.84, *p* = 0.404, Cohen's *d* = 0.14, *BF*_(01)_ = 4.13] or loss trials [*t*_(148)_ = 0.08, *p* = 0.938, Cohen's *d* = 0.01, *BF*_(01)_ = 5.67]. Figure 3C illustrates these findings.

#### 3.2.4. SMI

A 2 (incentivize) × 2 (task) ANOVA was conducted with SMI as a dependent variable. The results revealed a significant effect of task [*F*_(1, 148)_ = 25.43, *p* < 0.001, $\eta_{p}^{2}$ = 0.15], indicating that the SMI in the gain task (*M* = 0.80, 95% CI = [0.72, 0.87]) was significantly greater than that in the loss task (*M* = 0.62, 95% CI = [0.53, 0.70]). The main effect of incentivize was not significant [*F*_(1, 148)_ = 0.27, *p* = 0.603, $\eta_{p}^{2}$ = 0.004], and the interaction of incentive and task was not significant [*F*_(1, 148)_ = 0.55, *p* = 0.461, $\eta_{p}^{2}$ = 0.004]. Planned *t*-tests also showed no significant difference between incentivized and non-incentivized conditions in gain trials [*t*_(148)_ = 0.86, *p* = 0.390, Cohen's *d* = 0.14, *BF*_(01)_ = 4.04] or loss trials [*t*_(148)_ = 0.13, *p* = 0.900, Cohen's *d* = 0.02, *BF*_(01)_ = 5.65]. Figure 3D illustrates these findings.

## 4. Discussion

This study examined the effect of the incentive approach on intertemporal choice and found no significant difference in the proportion of choosing LL options between incentivized and non-incentivized intertemporal rewards. This coincides with previous research (Johnson and Bickel, 2002; Madden et al., 2003, 2004) and suggests that people discount hypothetical and real rewards similarly, and hypothetical rewards can be used as a valid proxy in research involving intertemporal choice. However, participants in the incentivized group exhibited more patience than those in the non-incentivized group in the loss task, although the evidence is weak. One explanation for this is loss aversion, where losses loom larger than gains (Loewenstein and Prelec, 1992; Scholten and Read, 2010). Recent evidence from neuroscience also indicate that the gains and losses domains of temporal discounting are underlined by two different intrinsic value systems (Zhang et al., 2018). Hence, when examining intertemporal choice in the loss domain, incentives become an unignorable and important issue.

We found that the participants' decision confidence increased when they were incentivized in gain and loss tasks, implying that participants were more certain of their decisions when they knew that their choices would be played for real. Recently, several studies have attempted to examine the confidence of intertemporal choice. For instance, Bulley et al. (2021) failed to observe that participants' confidence was higher when they chose the LL options, contrary to the assumptions of the self-control account. However, in their experiment, they used hypothetical rewards without incentive, potentially preventing them from finding the evidence supporting their hypothesis. These findings suggest that researchers should pay particular attention to incentives when examining the decision confidence of intertemporal choice.

Another finding of the present study is that the MFD was shorter in the incentivized group than in the non-incentivized group. The value of MFD is usually related to individuals' cognitive effort (Amblee et al., 2017) or the complexity level of information processing (Velichkovsky, 1999; Velichkovsky et al., 2002). Longer fixations are associated with deeper processing, such as deliberate consideration of information, whereas shorter fixations are associated with more superficial levels of processing (Glöckner and Herbold, 2011). Generally, the mean fixation duration of the deliberative strategy should be longer than that of the intuitive strategy (Glöckner and Herbold, 2011; Su et al., 2013). Results herein revealed that the mean fixation duration decreased when participants were incentivized, implying that the incentives reduce the complexity level of information processing and promote the intuitive decision strategy in intertemporal choice.

However, the effect of incentives on the values of TDT, OGP, and SMI remains unobserved. The values of TDT reflect the efficiency of information processing (Karalunas et al., 2012), OGP reflect the decision weight on outcome attribute (Zhou et al., 2021), and SMI reflect the direction of information search (i.e., alternative-wise vs. attribute-wise) (Su et al., 2013; Liu et al., 2021b; Zhou et al., 2022). Findings herein suggest that the incentives do not affect the key variables concerned by eye-tracking research on decision-making, such as decision weight and direction of information search.

The systematic variations between the gain and loss tasks was also obtained. Compared to participants in the gain task, those in the loss task showed more patience, shorter response time, greater decision confidence. The eye-tracking results showed that the outcome attribute received more attention and the information search was more attribute-wise in the loss task than the gain task. Following prior research on intertemporal choice (Thaler, 1981; Sun et al., 2015), our findings likewise suggest that the asymmetry between gain and loss can also be reflected in decision confidence and information processing. Although not the scope of the current study, future studies should further investigate the difference in the underlying mechanism between the gain and loss frame.

Some limitations were also noted herein. First, in the incentivized group, instead of getting the total of all results, participants were ultimately paid based on one randomly chosen trial. Previous research compared the pay-one condition and pay-all condition in the field of risky decision-making and shown that participants in the pay-all condition made 10% more riskier choices than those in other condition (Schmidt and Hewig, 2015). Future research may further compare the pay-one and pay-all conditions in intertemporal choice. Second, the outcomes and delays of the stimuli in the study herein were relatively small because of the cost. Previous research revealed that the magnitude of the outcomes and the delays could affect people's patience (Thaler, 1981; Loewenstein and Prelec, 1992). Future studies may examine the effect of incentives on intertemporal choice in larger magnitude options.

Ultimately, this study parallels the results of previous findings which posit that incentives do not affect intertemporal choice in the gain domain. Contrastingly, we found that incentives in the loss domain influenced people's temporal discounting. Furthermore, the decision confidence and the mean fixation duration differed between the incentivized and non-incentivized groups. These findings allow for a better understanding of the effect of incentives on intertemporal choice and provide valuable information for the design of incentives in future intertemporal experiments.

## Data availability statement

The datasets for this study can be found in the https://osf.io/prcbm/.

## Ethics statement

The studies involving human participants were reviewed and approved by Institutional Review Board of Nankai University. The patients/participants provided their written informed consent to participate in this study.

## Author contributions

X-LY and H-ZL conceived and designed this study, wrote the paper, and analyzed data. X-LY, S-TC, and H-ZL designed experimental stimuli and procedures. S-TC implemented experimental protocols and collected data. All authors contributed to the article and approved the submitted version.

## Funding

This work was partially supported by the National Natural Science Foundation of China (No. 71901126), the Humanity and Social Science Youth Foundation of Ministry of Education of China (No. 19YJC190013), and the Fundamental Research Funds for the Central Universities (No. 63222045).

## Conflict of interest

The authors declare that the research was conducted in the absence of any commercial or financial relationships that could be construed as a potential conflict of interest.

## Publisher's note

All claims expressed in this article are solely those of the authors and do not necessarily represent those of their affiliated organizations, or those of the publisher, the editors and the reviewers. Any product that may be evaluated in this article, or claim that may be made by its manufacturer, is not guaranteed or endorsed by the publisher.

## References

[B1] Ainslie G. (1975). Specious reward: a behavioral theory of impulsiveness and impulse control. Psychol. Bull. 82, 463–496. 10.1037/h0076860 1099599

[B2] Amasino D. R. Sullivan N. J. Kranton R. E. Huettel S. A. (2019). Amount and time exert independent influences on intertemporal choice. Nat. Hum. Behav. 3, 383–392. 10.1038/s41562-019-0537-2 30971787 PMC8020819

[B3] Amblee N. Ullah R. Kim W. (2017). Do product reviews really reduce search costs? J. Organ. Comput. Electron. Commerce 27, 199–217. 10.1080/10919392.2017.1332142

[B4] Ashby N. J. S. Johnson J. G. Krajbich I. Wedel M. (2016). Applications and innovations of eye-movement research in judgment and decision making. J. Behav. Decis. Mak. 29, 96–102. 10.1002/bdm.1956

[B5] Bickel W. K. Pitcock J. A. Yi R. Angtuaco E. J. (2009). Congruence of bold response across intertemporal choice conditions: fictive and real money gains and losses. J. Neurosci. 29, 8839–8846. 10.1523/JNEUROSCI.5319-08.2009 19587291 PMC2749994

[B6] Böckenholt U. Hynan L. S. (1994). Caveats on a process-tracing measure and a remedy. J. Behav. Decis. Mak. 7, 103–117. 10.1002/bdm.3960070203

[B7] Bulley A. Lempert K. M. Conwell C. Irish M. (2021). Intertemporal choice reflects value comparison rather than self-control: insights from confidence judgments. PsyArXiv. 10.31234/osf.io/w5zuk PMC9619231 36314145

[B8] Calluso C. Tosoni A. Cannito L. Committeri (2019). Concreteness and emotional valence of episodic future thinking (eft) independently affect the dynamics of intertemporal decisions. PLoS ONE 14, e0217224. 10.1371/journal.pone.0217224 31136620 PMC6538244

[B9] Cubitt R. McDonald R. Read D. (2017). Time matters less when outcomes differ: unimodal vs. cross-modal comparisons in intertemporal choice. Manag. Sci. 64, 873–887. 10.1287/mnsc.2016.2613

[B10] Dai J. Busemeyer J. R. (2014). A probabilistic, dynamic, and attribute-wise model of intertemporal choice. J. Exp. Psychol. Gen. 143, 1489–1514. 10.1037/a0035976 24635188 PMC4115005

[B11] Estle S. J. Green L. Myerson J. Holt D. D. (2007). Discounting of monetary and directly consumable rewards. Psychol. Sci. 18, 58–63. 10.1111/j.1467-9280.2007.01849.x 17362379

[B12] Faul F. Erdfelder E. Lang A. G. Buchner A. (2007). G*power 3: A flexible statistical power analysis program for the social, behavioral, and biomedical sciences. Behav. Res. Methods 39, 175–191. 10.3758/BF03193146 17695343

[B13] Fisher G. (2021). Intertemporal choices are causally influenced by fluctuations in visual attention. Manag. Sci. 67, 4961–4981. 10.1287/mnsc.2020.3732

[B14] Fleming S. M. Dolan R. J. Frith C. D. (2012). Metacognition: computation, biology and function. R. Soc. 367, 1280–1286. 10.1098/rstb.2012.0021 22492746 PMC3318771

[B15] Fleming S. M. Lau H. C. (2014). How to measure metacognition. Front. Hum. Neurosci. 8, 443. 10.3389/fnhum.2014.00443 25076880 PMC4097944

[B16] Franco-Watkins A. M. Mattson R. E. Jackson M. D. (2016). Now or later? attentional processing and intertemporal choice. J. Behav. Decis. Mak. 29, 206–217. 10.1002/bdm.1895

[B17] Frederick S. Loewenstein G. O'Donoghue T. (2002). Time discounting and time preference: a critical review. J. Econ. Lit. 40, 351–401. 10.1257/jel.40.2.35126063653

[B18] Glaholt M. G. Reingold E. M. (2011). Eye movement monitoring as a process tracing methodology in decision making research. J. Neurosci. Psychol. Econ. 4, 125–146. 10.1037/a0020692

[B19] Glöckner A. Herbold A. (2011). An eye-tracking study on information processing in risky decisions: evidence for compensatory strategies based on automatic processes. J. Behav. Decis. Mak. 24, 71–98. 10.1002/bdm.684

[B20] Hardisty D. J. Weber E. U. (2009). Discounting future green: money versus the environment. J. Exp. Psychol. Gen. 138, 329–340. 10.1037/a0016433 19653793

[B21] Harris C. R. (2012). Feelings of dread and intertemporal choice. J. Behav. Decis. Mak. 25, 13–28. 10.1002/bdm.709

[B22] Horstmann N. Ahlgrimm A. Glöckner A. (2009). How distinct are intuition and deliberation? an eye-tracking analysis of instruction-induced decision modes. Judgm. Decis. Mak. 4, 335–354. 10.2139/ssrn.1393729

[B23] Jiang Q. Liu L. (2021). Temporal course of the sign effect in intertemporal choice. J. Cogn. Psychol. 33, 595–607. 10.1080/20445911.2021.1948551

[B24] Johnson M. W. Bickel W. K. (2002). Within-subject comparison of real and hypothetical money rewards in delay discounting. J. Exp. Anal. Behav. 77, 129–146. 10.1901/jeab.2002.77-129 11936247 PMC1284852

[B25] Karalunas S. L. Huang-Pollock C. L. Nigg J. T. (2012). Decomposing attention-deficit/hyperactivity disorder (adhd)-related effects in response speed and variability. Neuropsychology 26, 684–694. 10.1037/a0029936 23106115 PMC3516369

[B26] Kirby K. N. (1997). Bidding on the future: evidence against normative discounting of delayed rewards. J. Exp. Psychol. Gen. 126, 54–70. 10.1037/0096-3445.126.1.54

[B27] Krajbich I. Bartling B. Hare T. Fehr E. (2015). Rethinking fast and slow based on a critique of reaction-time reverse inference. Nat. Commun. 6, 1–9. 10.1038/ncomms8455 26135809 PMC4500827

[B28] Lagorio C. H. Madden G. J. (2005). Delay discounting of real and hypothetical rewards III: steady-state assessments, forced-choice trials, and all real rewards. Behav. Processes 69, 173–187. 10.1016/j.beproc.2005.02.003 15845306

[B29] Lak A. Costa G. M. Romberg E. Koulakov A. A. Mainen Z. F. Kepecs A. (2014). Orbitofrontal cortex is required for optimal waiting based on decision confidence. Neuron 84, 190–201. 10.1016/j.neuron.2014.08.039 25242219 PMC4364549

[B30] Liu H. Z. Lyu X. K. Wei Z. H. Mo W. L. Luo J. R. Su X. Y. (2021a). Exploiting the dynamics of eye gaze to bias intertemporal choice. J. Behav. Decis. Mak. 34, 419–431. 10.1002/bdm.2219

[B31] Liu H. Z. Wei Z. H. Li P. (2021b). Influence of the manner of information presentation on risky choice. Front. Psychol. 12. 10.3389/fpsyg.2021.650206 34759853 PMC8573322

[B32] Loewenstein G. Prelec D. (1992). Anomalies in intertemporal choice: evidence and an interpretation. Q. J. Econ. 107, 573–597. 10.2307/2118482

[B33] Madden G. J. Begotka A. M. Raiff B. R. Kastern L. L. (2003). Delay discounting of real and hypothetical rewards. Exp. Clin. Psychopharmacol. 11, 139–145. 10.1037/1064-1297.11.2.139 12755458

[B34] Madden G. J. Raiff B. R. Lagorio C. H. Begotka A. M. Mueller A. M. Hehli D. J.. (2004). Delay discounting of potentially real and hypothetical rewards: II. Between-and within-subject comparisons. Exp. Clin. Psychopharmacol. 12, 251–261. 10.1037/1064-1297.12.4.251 15571442

[B35] Marini M. Ansani A. Paglieri F. (2020). Attraction comes from many sources: attentional and comparative processes in decoy effects. Judgm. Decis. Mak. 15, 704–726.

[B36] Pachur T. Hertwig R. Gigerenzer G. Brandstätter E. (2013). Testing process predictions of models of risky choice: a quantitative model comparison approach. Front. Psychol. 4, 646. 10.3389/fpsyg.2013.00646 24151472 PMC3784771

[B37] Peterson D. K. Pitz G. F. (1988). Confidence, uncertainty, and the use of information. J. Exp. Psychol. Learn. Mem. Cogn. 14, 85–92. 10.1037/0278-7393.14.1.85

[B38] Rao L. L. Li S. (2011). New paradoxes in intertemporal choice. Judgm. Decis. Mak. 6, 122–129.

[B39] Rayner K. (1998). Eye movements in reading and information processing: 20 years of research. Psychol. Bull. 124, 372–422. 10.1037/0033-2909.124.3.372 9849112

[B40] Rayner K. (2009). Eye movements and attention in reading, scene perception, and visual search. Q. J. Exp. Psychol. 62, 1457–1506. 10.1080/17470210902816461 19449261

[B41] Rayner K. Chase K. H. Slattery T. J. Ashby J. (2006). Eye movements as reflections of comprehension processes in reading. Sci. Stud. Read. 10, 241–255. 10.1207/s1532799xssr1003_3

[B42] Read D. Frederick S. Scholten M. (2013). Drift: an analysis of outcome framing in intertemporal choice. J. Exp. Psychol. Learn. Mem. Cogn. 39, 573–588. 10.1037/a0029177 22866891

[B43] Reeck C. Wall D. Johnson E. J. (2017). Search predicts and changes patience in intertemporal choice. Proc. Natl. Acad. Sci. U.S.A. 114, 11890–11895. 10.1073/pnas.1707040114 29078303 PMC5692544

[B44] Rubinstein A. (2007). Instinctive and cognitive reasoning: a study of response times. Econ. J. 117, 1243–1259. 10.1111/j.1468-0297.2007.02081.x

[B45] Şahin M. Aybek E. (2019). Jamovi: an easy to use statistical software for the social scientists. Int. J. Assess. Tools Educ. 6, 670–692. 10.21449/ijate.661803

[B46] Samuelson P. A. (1937). A note on measurement of utility. Rev. Econ. Stud. 4, 155–161. 10.2307/2967612

[B47] Sandberg K. Timmermans B. Overgaard M. Cleeremans A. (2010). Measuring consciousness: is one measure better than the other? Conscious Cogn. 19, 1069–1078. 10.1016/j.concog.2009.12.013 20133167

[B48] Schmidt B. Hewig J. (2015). Paying out one or all trials: a behavioral economic evaluation of payment methods in a prototypical risky decision study. Psychol. Rec. 65, 245–250. 10.1007/s40732-014-0112-1

[B49] Scholten M. Read D. (2010). The psychology of intertemporal tradeoffs. Psychol. Rev. 117, 925–944. 10.1037/a0019619 20658858

[B50] Stewart N. Hermens F. Matthews W. J. (2015). Eye movements in risky choice. J. Behav. Decis. Mak. 29, 116–136. 10.1002/bdm.1854 27522985 PMC4964953

[B51] Su Y. Sun L. L. Du H. Y. Xue L. Li X. S. Li S. (2013). Is making a risky choice based on a weighting and adding process? an eye-tracking investigation. J. Exp. Psychol. Learn. Mem. Cogn. 39, 1765–1780. 10.1037/a0032861 23687917

[B52] Sun H. Y. Li A. M. Chen S. Zhao D. Rao L. L. Liang Z. Y.. (2015). Pain now or later: An outgrowth account of pain-minimization. PLoS ONE 10, e0119320. 10.1371/journal.pone.0119320 25747461 PMC4352049

[B53] Thaler R. (1981). Some empirical evidence on dynamic inconsistency. Econ. Lett. 8, 201–207. 10.1016/0165-1765(81)90067-7

[B54] van Doorn J. van den Bergh D. Bohm U. Dablander F. Derks K. Draws T.. (2021). The jasp guidelines for conducting and reporting a bayesian analysis. Psychon. Bull. Rev. 28, 813–826. 10.3758/s13423-020-01798-5 33037582 PMC8219590

[B55] Velichkovsky B. M. (1999). “From levels of processing to stratification of cognition: Converging evidence from three domain of research,” in Stratification in Cognition and Consciousness, eds Challis, B. H. and Velichkosky B. M. (Philadelphia, PA: John Benjamins Publishing Company) 203–235.

[B56] Velichkovsky B. M. Rothert A. Kopf M. Dornhöfer S. M. Joos M. (2002). Towards an express-diagnostics for level of processing and hazard perception. Transport. Res. F 5, 145–156. 10.1016/S1369-8478(02)00013-X

[B57] Wagenmakers E. J. Marsman M. Jamil T. Ly A. Verhagen J. Love J.. (2018). Bayesian inference for psychology. Part i: Theoretical advantages and practical ramifications. Psychon. Bull. Rev. 25, 35–57. 10.3758/s13423-017-1343-3 28779455 PMC5862936

[B58] Wang Q. Yang S. Liu M. Cao Z. Ma Q. (2014). An eye-tracking study of website complexity from cognitive load perspective. Decis. Support Syst. 62, 1–10. 10.1016/j.dss.2014.02.007

[B59] Weber E. U. Johnson E. J. Milch K. F. Chang H. Brodscholl J. C. Goldstein D. G. (2007). Asymmetric discounting in intertemporal choice: a query-theory account. Psychol. Sci. 18, 516–523. 10.1111/j.1467-9280.2007.01932.x 17576265

[B60] Wierzchon M. Asanowicz D. Paulewicz B. Cleeremans A. (2012). Subjective measures of consciousness in artificial grammar learning task. Conscious Cogn. 21, 1141–1153. 10.1016/j.concog.2012.05.012 22728143

[B61] Zhang Y. Y. Xu L. J. Liang Z. Y. Wang K. Hou B. Zhou Y.. (2018). Separate neural networks for gains and losses in intertemporal choice. Neurosci. Bull. 34, 725–735. 10.1007/s12264-018-0267-x 30088149 PMC6129240

[B62] Zhou Y. B. Li Q. Li Q. Y. Liu H. Z. (2022). Evaluation scale or output format: the attentional mechanism underpinning time preference reversal. Front. Psychol. 13, 865598. 10.3389/fpsyg.2022.865598 35496199 PMC9046692

[B63] Zhou Y. B. Li Q. Liu H. Z. (2021). Visual attention and time preference reversals. Judgm. Decis. Mak. 16, 1010–1038.35496199

